# Impact of Smear Layer Removal Using Etidronate- and EDTA-Based Irrigation Protocols on Root Canal Microbiota: An In Vivo Study

**DOI:** 10.3390/dj14030151

**Published:** 2026-03-07

**Authors:** Svetlana Razumova, Anzhela Brago, Anzhelika Kryuchkova, Zilolakhon Khakimova, Nazira Khabibova, Alexander Volkov, Haydar Barakat, Natalya Dikopova

**Affiliations:** 1 Department of Propaedeutics of Dental Diseases, Medical Institute, Patrice Lumumba Peoples’ Friendship University of Russia (RUDN University), 6 Miklukho-Maklaya Street, Moscow 117198, Russia; razumova-sn@rudn.ru (S.R.); brago-as@rudn.ru (A.B.); kryuchkova-av@rudn.ru (A.K.); 2 Department of Propaedeutics Dentistry, Andijan State Medical Institute, Yu.Otabekov 1, Andijan City 170119, Uzbekistan; lola-zilola@mail.ru; 3 Department of Conservative Dentistry, Bukhara State Medical Institute Named After Abu Ali ibn Sino, Bukhara 200126, Uzbekistan; nazira.habibova@bsmi.uz; 4 Department of Therapeutic Dentistry, E.V. Borovsky Institute of Dentistry, FSAEI HE I.M. Sechenov First MSMU of MOH of Russia, Sechenovskiy University, 8/2 Trubetskaya St., Moscow 119991, Russia; parodont@inbox.ru (A.V.); zubnoy-doctor@yandex.ru (N.D.)

**Keywords:** chronic apical periodontitis, root canal, smear layer, irrigation, etidronate, root canal microbiota

## Abstract

**Background**: The effective elimination of root canal microbiota is essential for the treatment of apical periodontitis. The smear layer formed during instrumentation limits the penetration of irrigants into dentinal tubules, making chelation a critical component of irrigation protocols. While ethylenediaminetetraacetic acid (EDTA) is commonly used for smear layer removal, etidronate has been proposed as an alternative due to its chemical compatibility with sodium hypochlorite. The aim of this study was to compare the effectiveness of different irrigation protocols in eliminating microorganisms from the root canal system in patients with chronic apical periodontitis. **Methods**: Thirty patients aged 18–44 years diagnosed with chronic apical periodontitis (ICD-10 code K04.5) were included. Three irrigation protocols were evaluated: (1) 3% sodium hypochlorite followed by 17% EDTA; (2) 3% sodium hypochlorite followed by a 9% aqueous solution of etidronate; and (3) a 9% solution of etidronate dissolved in 3% sodium hypochlorite (continuous chelation). Microbiological samples were collected before and after root canal instrumentation and irrigation. Microbial analysis was performed using gas chromatography–mass spectrometry. **Results**: All protocols resulted in a reduction in microbial load. However, protocols using 3% sodium hypochlorite with 17% EDTA and continuous chelation with etidronate in sodium hypochlorite demonstrated a greater number of statistically significant reductions. Sequential irrigation with aqueous etidronate showed the lowest antimicrobial effectiveness. **Conclusions**: Continuous chelation with etidronate in 3% sodium hypochlorite showed promising antimicrobial performance and may represent a clinically feasible alternative irrigation strategy.

## 1. Introduction

Apical periodontitis is a disease characterized by the destruction of periodontal tissues resulting from pulp necrosis and subsequent colonization of the root canal system by microorganisms [1]. The primary objective of endodontic treatment is to reduce the microbial load below a critical threshold required to initiate and maintain periapical healing [2]. Reduction in root canal microbiota is achieved through a combination of mechanical instrumentation and chemical irrigation [3].

Stainless steel and nickel–titanium endodontic instruments are routinely used for canal preparation; they remove infected dentin and facilitate irrigant penetration [4]. However, due to the complex anatomy of the root canal system, a substantial portion of the canal walls often remains uninstrumented following mechanical preparation alone. Consequently, various irrigation strategies and activation techniques have been developed to enhance canal disinfection [5,6,7].

Irrigating solutions address two principal challenges associated with endodontic instrumentation: dissolution of residual organic tissue and removal of inorganic dentin debris generated during canal preparation. Sodium hypochlorite (NaOCl), used at concentrations of 1–5%, remains the most widely applied irrigant for pulp tissue dissolution and antimicrobial disinfection [8]. Its antimicrobial efficacy is well documented, including activity against biofilm-associated microorganisms [9,10].

Removal of the inorganic component of dentin debris and the smear layer is achieved using chelating agents. Ethylenediaminetetraacetic acid (EDTA) at concentrations of 17–20% is the conventional agent for smear layer removal and is typically applied sequentially with sodium hypochlorite or as a final irrigant [11]. This combination facilitates elimination of tissue remnants that serve as nutrient substrates for microorganisms, improves irrigant penetration into dentinal tubules and isthmuses, and enhances access to anatomically complex regions of the root canal system.

A major limitation of this protocol is the potential chemical interaction between EDTA and sodium hypochlorite when the solutions come into direct contact within the root canal, potentially reducing available chlorine and altering chelation effectiveness; the magnitude of this effect is dependent on clinical variables such as irrigant volumes, contact time, and whether intermediate flushing is used [12].

An alternative approach involves the use of chelating agents that preserve the chemical stability and antibacterial properties of sodium hypochlorite while enabling continuous chelation. Sodium etidronate has been proposed for this purpose. Etidronate, a salt of etidronic acid, is a water-soluble compound with acidic properties and an overall alkaline pH. Its lower affinity for calcium compared with EDTA makes it less aggressive toward dentin, reducing the risk of erosion even during prolonged intracanal exposure [13]. The lower calcium affinity of etidronate compared with EDTA is clinically relevant because it reduces the risk of aggressive dentin erosion during longer exposure while still providing chelation. Importantly, etidronate can be mixed with NaOCl to enable continuous chelation with documented chemical compatibility [9,12].

Etidronate may be applied either as an aqueous solution in sequential irrigation protocols or mixed directly with sodium hypochlorite. When dissolved in NaOCl, etidronate can be used as a single irrigant throughout all stages of canal preparation, providing continuous chelation without compromising antimicrobial efficacy [14,15].

Effective smear layer removal along the entire length of the root canal, including isthmuses and canal invaginations, may positively influence treatment outcomes in chronic apical periodontitis [16]. Ex vivo SEM studies suggest that etidronate-based protocols can improve smear layer removal, including in the apical third, and activation may enhance this effect; however, clinical effectiveness depends on irrigant delivery and canal anatomy [15]. Enhanced contact between antiseptic irrigants and dentin surfaces facilitates microbial elimination; however, microorganisms may penetrate dentinal tubules to depths of up to 200 μm, complicating disinfection [17,18].

The microbial composition of root canals in apical periodontitis is influenced by disease duration, communication with the oral cavity, and the predominantly anaerobic environment of the canal system. These factors promote the formation of structured bacterial biofilms, which increase microbial resistance to antiseptics and may serve as a source of secondary contamination if preserved beneath the smear layer [19]. According to Caldwell, biofilms exhibit defining characteristics including autopoiesis, communal behavior, synergy, and homeostasis [20].

Root canal biofilms commonly comprise cariogenic microorganisms and periodontal pathogens, such as Streptococcus mutans, *Aggregatibacter actinomycetemcomitans*, and *Porphyromonas gingivalis*, as well as species adapted to anaerobic conditions, notably *Enterococcus faecalis* [21]. Given the prominent role of *E. faecalis* in persistent endodontic infections, the ability of irrigants to disrupt biofilm structure is of particular importance [22].

Irrigants used in the treatment of chronic apical periodontitis should therefore exhibit surfactant properties, enabling disruption of interbacterial bonds within biofilms and improving antiseptic penetration. Both EDTA and etidronate solutions possess surfactant characteristics, which may enhance their antimicrobial effectiveness when combined with sodium hypochlorite. The Aim of the Study was to compare the effectiveness of different irrigation protocols in eliminating microorganisms from the root canal system in patients with chronic apical periodontitis.

## 2. Materials and Methods

### 2.1. Study Design and Ethical Approval

This prospective clinical study was conducted at the Dental Clinics of the Peoples’ Friendship University of Russia (RUDN University), Moscow, Russian Federation. The study protocol was reviewed and approved by the Local Ethics Committee of RUDN University (approval No. 21/19 October 2023). All procedures were performed in accordance with the principles of the Declaration of Helsinki.

Prior to participation, all patients were informed about the nature of the study and the differences between irrigation protocols. Written informed consent was obtained from all participants before enrollment.

### 2.2. Study Population

The study included 30 patients diagnosed with chronic apical periodontitis (ICD-10 code K04.5). Patients of both sexes aged 18–44 years were enrolled. Subjects were randomly assigned to one of three treatment groups. Each patient contributed one tooth to the study.

### 2.3. Endodontic Treatment Protocol

All endodontic procedures were performed under standardized clinical conditions. Mechanical and chemical preparation of the root canals was carried out according to the assigned irrigation protocol. After completion of canal preparation, calcium hydroxide was placed as an intracanal medicament. Root canal obturation was performed at a second visit.

- Operative Field Preparation and Decontamination

Prior to treatment, supragingival plaque was removed using an air-polishing device. Caries excavation was performed before rubber dam placement. After rubber dam isolation, pre-endodontic restoration with composite material was carried out when required to ensure adequate coronal sealing. The operative field and access cavity area were subsequently disinfected with 3% sodium hypochlorite before access to the root canal system was established.

B. Patient and Tooth Selection Criteria

The study included patients diagnosed with pulp necrosis and apical periodontitis who had not previously undergone endodontic treatment. Both primary carious lesions and teeth previously restored for caries were included. All teeth had sufficient remaining tooth structure to allow adequate future prosthetic rehabilitation and showed no visible cracks. The absence of cracks was verified visually after complete removal of existing restorations using methylene blue staining. Teeth with signs of periodontal disease were excluded.

C. Preparation of Etidronate–NaOCl Solution and Irrigant Volume

Etidronate powder (VladMiVa, Belgorod, Russia) supplied in pre-measured 0.9 g glass vials was used for solution preparation in accordance with the manufacturer’s instructions. For each preparation, 0.9 g of etidronate powder was dissolved in 9.1 mL of 3% sodium hypochlorite, measured using a sterile disposable syringe. The solution was used after the complete cessation of gas release.

During instrumentation, approximately 1.8 mL of the prepared etidronate–NaOCl solution was delivered after each instrument.

### 2.4. Group Allocation and Irrigation Protocols

Included teeth comprised incisors, canines, premolars, and molars; distribution by group is detailed below.

- Group 1

Ten teeth (1 incisor, 3 premolars, and 6 molars) were instrumented using C-Flexi instruments (Geosoft, Moscow, Russia) up to an apical master file size #35. Irrigation was performed according to Protocol 1 using a syringe and endodontic irrigation needle positioned 1–2 mm short of the established working length with 3% sodium hypochlorite (NaOCl—1.8 mL) and 17% EDTA (2 mL) after each endodontic instrument. EDTA and NaOCl were activated for 30 s using an EQ-S endoactivator (Meta Biomed, Chong Ju City, Republic of Korea). Between irrigants, the root canal was flushed with sterile water to prevent chemical interaction.

- Group 2

Ten teeth (3 incisors, 1 canine, 4 premolars, and 2 molars) were prepared using C-Flexi instruments up to an apical master file size #35. Irrigation followed Protocol 2, involving sequential irrigation with 3% NaOCl (1.8 mL) and a 9% aqueous solution of etidronate (0.9 g etidronate dissolved in 9.1 mL sterile water) (1.8 mL) after each instrument. The 9% etidronate solution was activated for 30 s using the EQ-S endoactivator. No intermediate water irrigation was performed between solutions.

- Group 3

Ten teeth (6 incisors, 1 canine, and 3 premolars) were instrumented using C-Flexi instruments up to an apical master file size #35. Irrigation was performed according to Protocol 3, which involved continuous chelation with a 9% solution of etidronate in 3% sodium hypochlorite (0.9 g etidronate dissolved in 9.1 mL of 3% NaOCl) (1.8 mL). After each instrument, the solution was activated for 30 s using the EQ-S endoactivator.

Before post-instrumentation sampling, the canals were dried under standardized conditions using sterile paper points.

### 2.5. Microbiological Sampling and Analysis

Microbiological samples were collected in each group during the first visit using sterile K-files. Samples were obtained before mechanical and chemical preparation and after completion of treatment according to the study protocol. All samples were fixed, assigned coded identifiers, and transported to the laboratory for microbiological analysis.

Microbiological analysis was performed using gas chromatography–mass spectrometry (GC–MS) with the MAESTRO GC system. This method is based on chromatographic and mass spectrometric analysis of microbial cell lipid composition. Qualitative and quantitative assessment of specific fatty acids and aldehydes present in membrane lipids allows the generation of determinant profiles used for differential diagnosis and identification of microorganisms.

### 2.6. Statistical Analysis

For statistical evaluation, microorganisms most frequently detected across all 30 samples were selected to ensure data completeness. Statistical analysis was conducted using standard methods in a Windows 11 environment with Statistica 10 and SAS JMP 11 software (SAS Institute Inc., Cary, NC, USA). Changes in microbial counts before and after treatment were analyzed using the nonparametric Wilcoxon signed-rank test. A *p*-value < 0.05 was considered statistically significant.

## 3. Results

At the first stage of analysis, the dominant microorganisms were identified in all pre-treatment (“Before”) samples, and the data were subjected to statistical analysis. At the subsequent stage, post-treatment results obtained for all irrigation protocols were statistically processed.

Within-protocol comparisons of microbial counts in “Before” and “After” samples were then performed separately for each irrigation protocol. The results of the statistical analysis are presented in Table 1, Table 2 and Table 3, where M represents the mean value, SD the standard deviation, changes in mean values between “Before” and “After” samples are expressed as percentages, and statistical significance at *p* < 0.05 is indicated by an asterisk (*).

According to the data presented in Table 1, Table 2 and Table 3, all irrigation protocols demonstrated a reduction in the total number of microorganisms in post-treatment samples compared with baseline values. The magnitude of microbial reduction across the three protocols ranged from −45.9% to −100%. The lowest overall reduction trend was observed in Protocol 2 (sequential irrigation with 3% sodium hypochlorite and 9% etidronate in sterile water), in which an increase in Streptomyces spp. was observed, with a mean increase of 41.2% (Table 2).

In Protocol 1 (sequential irrigation with 3% sodium hypochlorite and 17% EDTA), a consistent reduction in microbial counts was observed, ranging from −81.0% to −99.4%. Statistically significant differences between “Before” and “After” samples were detected for five taxa: *Lactobacillus* spp., *Propionibacterium* spp., *Peptostreptococcus anaerobius* strain 18623, *Rhodococcus* spp., and *Streptomyces* spp. Notably, none of the investigated microorganisms were completely eliminated in this group (Table 1).

In Protocol 3, which involved continuous irrigation with a 9% solution of etidronate in 3% sodium hypochlorite, the reduction dynamics ranged from −77.4% to −100%. Complete elimination of microorganisms in post-treatment samples was observed for three taxa: *Peptostreptococcus anaerobius* strain 18623, *Clostridium perfringens*, and *Rhodococcus* spp. In addition, four taxa, *Bacillus megaterium*, *Lactobacillus* spp., *Propionibacterium* spp., and *Corynebacterium* spp. demonstrated statistically significant reductions in mean concentrations (Table 3).

## 4. Discussion

Effective elimination of microorganisms from the root canal system remains a central challenge in endodontic therapy, particularly in the presence of complex canal anatomy and biofilm-associated infections. While smear layer removal is widely recognized as a prerequisite for adequate disinfection, the clinical antimicrobial consequences of different chelation strategies remain insufficiently explored in vivo. In this context, the present study provides clinically relevant evidence by comparing the effects of EDTA- and etidronate-based irrigation protocols on root canal microbiota under real clinical conditions. The findings contribute to a more precise understanding of how chelator selection and irrigation sequence influence antimicrobial efficacy.

In our study, protocol 2, which involved sequential irrigation with 3% sodium hypochlorite and 9% etidronate in sterile water, demonstrated the lowest antimicrobial efficacy among the evaluated protocols. This outcome may be explained by the dilution of sodium hypochlorite by the water-based chelating solution. Previous studies have shown that etidronate dissolved in distilled water effectively removes the smear layer by opening dentinal tubules and isthmuses. However, this process may also expose microorganisms previously protected beneath the smear layer, allowing them to enter the irrigant within the root canal and resulting in an apparent increase in bacterial counts. Simultaneously, dilution of sodium hypochlorite likely reduces its antimicrobial potency, rendering it insufficient to eliminate bacteria released from dentin debris.

Despite these short-term findings, removal of the smear layer may still offer benefits at later treatment stages. In particular, the absence of a smear layer may facilitate direct contact between calcium hydroxide and the root canal walls, potentially enhancing its antimicrobial action and reducing residual microbial contamination during interappointment medication.

In the group treated with sequential irrigation using 3% sodium hypochlorite and 17% EDTA (Protocol 1), a stable trend toward reduction in bacterial load was observed. However, these results should be interpreted with caution. Numerous studies have demonstrated the limited effectiveness of 17% EDTA in removing the smear layer in the apical third of the root canal [23,24]. As a result, antimicrobial activity may be restricted to superficial components of the smear layer, allowing microorganisms to persist within dentinal tubules and isthmuses and limiting the penetration of intracanal medicaments such as calcium hydroxide. Furthermore, extensive literature data indicate adverse effects of EDTA on root dentin structure, as well as a reduction in available chlorine when EDTA interacts with sodium hypochlorite, which further compromises antimicrobial efficacy [25]. These limitations prevent EDTA from being considered an optimal chelating agent in combined irrigation protocols.

In contrast, Protocol 3, which employed continuous chelation with a 9% solution of etidronate in 3% sodium hypochlorite, demonstrated stable and consistent microbial reduction, with no instances of increased microbial counts. According to previously published data, continuous chelation with etidronate enables effective smear layer removal along the entire length of the root canal system, including the apical region and isthmuses, and promotes antimicrobial action within dentinal tubules [15,26]. The results of the present study support the concept that dissolution of dry etidronate salt directly in sodium hypochlorite does not compromise its antimicrobial activity, while simultaneously enhancing smear layer removal. Similar observations have been reported by other authors, further confirming the advantages of continuous chelation strategies [27].

Overall, these findings highlight the clinical relevance of irrigation protocol design and underscore the importance of integrating smear layer removal with sustained antimicrobial activity to optimize root canal disinfection. Nevertheless, the present results should be interpreted as preliminary and hypothesis-generating rather than confirmatory.

The present study has several strengths that enhance its clinical relevance. First, the investigation was conducted in vivo under real clinical conditions, allowing assessment of irrigation protocols in a setting that closely reflects routine endodontic practice. Second, the study compared clinically applicable irrigation strategies, including continuous chelation with etidronate, using standardized instrumentation and activation protocols. In addition, application of gas chromatography–mass spectrometry enabled detailed qualitative and quantitative characterization of root canal microbiota beyond conventional culture-based methods.

At the same time, several limitations should be acknowledged. First, the present study should be considered exploratory in nature due to the relatively limited sample size, which restricts statistical power and generalizability. Although sufficient for preliminary in vivo assessment, the number of cases does not allow definitive conclusions regarding the superiority of one protocol over another, and larger multicenter studies are required to confirm these findings.

Second, unequal distribution of tooth types among groups represents a potential confounding factor, as anatomical complexity and variations in root canal morphology may influence irrigant penetration and microbial reduction. This factor may have contributed to interindividual variability in treatment outcomes.

Third, direct intergroup statistical comparisons were limited by sample size and variability and therefore were not included in the present analysis. Future studies with larger cohorts are needed to enable robust comparative statistical evaluation between irrigation protocols.

Finally, microbiological analysis based on GC–MS detects lipid biomarkers and does not differentiate between viable and non-viable microorganisms. Although this method provides a comprehensive overview of microbial profiles, complementary molecular techniques such as quantitative PCR or next-generation sequencing may offer additional information on bacterial viability and should be considered in future investigations. Furthermore, microbiological assessment was performed at defined treatment stages and did not include long-term clinical or radiographic follow-up.

## 5. Conclusions

Within the limitations of this in vivo study, continuous chelation with etidronate in 3% sodium hypochlorite (Protocol 3) showed promising and consistent antimicrobial effects against root canal microbiota. In contrast, sequential irrigation with 3% sodium hypochlorite and 9% etidronate in sterile water (Protocol 2) demonstrated lower and more variable antimicrobial performance. These findings suggest that combining sustained antimicrobial activity with effective smear layer removal may enhance root canal disinfection in the management of chronic apical periodontitis. However, due to the limited sample size, unequal distribution of tooth types, and absence of long-term clinical follow-up, definitive conclusions regarding the superiority of specific irrigation protocols cannot be drawn. Further well-designed, large-scale clinical studies are required to confirm these preliminary observations and to verify their long-term clinical relevance.

## Figures and Tables

**Table 1:** Comparison of mean microbial counts in “Before” and “After” samples using Protocol 1 (3% NaOCl + 17% EDTA).

Microorganism	M ± SD, Before	M ± SD, After	Mean Change	*p* Value
*Bacillus megaterium*	105.90 ± 196.68	2.10 ± 5.13	−98.0%	0.0679
*Lactobacillus* spp.	1380.50 ± 2071.73	8.70 ± 14.58	−99.4%	0.0180 *
*Propionibacterium* spp.	227.70 ± 406.24	4.10 ± 8.24	−98.2%	0.0280 *
*Peptostreptococcus anaerobius* 18623	669.50 ± 1319.98	24.70 ± 34.00	−96.3%	0.0464 *
*Clostridium perfringens*	1646.30 ± 3368.15	74.10 ± 186.59	−95.5%	0.1235
*Corynebacterium* spp.	99.60 ± 135.98	18.90 ± 27.08	−81.0%	0.0630
*Rhodococcus* spp.	26.70 ± 36.77	2.30 ± 4.00	−91.4%	0.0431 *
*Streptomyces* spp.	127.10 ± 229.77	3.40 ± 4.97	−97.3%	0.0464 *

Note: * *p* < 0.05, statistically significant difference between “Before” and “After” samples.

**Table 2:** Comparison of mean microbial counts in “Before” and “After” samples using Protocol 2 (3% NaOCl + 9% etidronate in sterile water).

Microorganism	M ± SD, Before	M ± SD, After	Mean Change	*p* Value
*Bacillus megaterium*	2.80 ± 5.92	1.10 ± 3.48	−60.7%	0.1797
*Lactobacillus* spp.	116.30 ± 173.71	26.70 ± 29.61	−77.0%	0.2135
*Propionibacterium* spp.	27.10 ± 39.44	3.20 ± 4.39	−88.2%	0.1141
*Peptostreptococcus anaerobius* 18623	29.40 ± 51.99	15.90 ± 26.15	−45.9%	0.1088
*Clostridium perfringens*	89.00 ± 189.28	1.50 ± 3.57	−98.3%	0.0464 *
*Corynebacterium* spp.	12.40 ± 19.77	3.70 ± 5.06	−70.2%	0.5940
*Rhodococcus* spp.	23.00 ± 48.82	2.30 ± 4.06	−90.0%	0.1056
*Streptomyces* spp.	3.40 ± 7.89	4.80 ± 8.77	+41.2%	0.7150

Note: * *p* < 0.05, statistically significant difference between “Before” and “After” samples.

**Table 3:** Comparison of mean microbial counts in “Before” and “After” samples using Protocol 3 (continuous chelation: 9% etidronate in 3% NaOCl).

Microorganism	M ± SD, Before	M ± SD, After	Mean Change	*p* Value
*Bacillus megaterium*	95.60 ± 214.74	1.10 ± 2.33	−98.9%	0.0431 *
*Lactobacillus* spp.	272.60 ± 701.43	4.60 ± 13.85	−98.3%	0.0209 *
*Propionibacterium* spp.	239.00 ± 550.55	1.60 ± 2.99	−99.3%	0.0277 *
*Peptostreptococcus anaerobius* 18623	540.90 ± 1710.48	0.00 ± 0.00	−100.0%	0.3173
*Clostridium perfringens*	1.20 ± 2.57	0.00 ± 0.00	−100.0%	0.1797
*Corynebacterium* spp.	12.40 ± 26.01	2.80 ± 4.57	−77.4%	0.0431 *
*Rhodococcus* spp.	73.60 ± 186.75	0.00 ± 0.00	−100.0%	0.1088
*Streptomyces* spp.	55.60 ± 148.90	1.90 ± 4.07	−96.6%	0.0679

Note: * *p* < 0.05, statistically significant difference between “Before” and “After” samples.

## Data Availability

The original contributions presented in this study are included in the article. Further inquiries can be directed to the corresponding author.
